# Neuromuscular activity of the lower-extremities during running, landing and changing-of-direction movements in individuals with anterior cruciate ligament reconstruction: a review of electromyographic studies

**DOI:** 10.1186/s40634-023-00603-1

**Published:** 2023-04-14

**Authors:** Jim D. Georgoulis, Dimitra Melissaridou, Kostas Patras, Panayiotis D. Megaloikonomos, Ioannis Trikoupis, Olga D. Savvidou, Panayiotis J. Papagelopoulos

**Affiliations:** 1 First Department of Orthopaedic Surgery, National and Kapodistrian University of Athens, Athens, Greece; 2 Orthopaedic Sports Medicine Center of Ioannina, University of Ioannina, Ioannina, Greece; 3 Pan Am Clinic, University of Manitoba, Winnipeg, MB Canada

**Keywords:** EMG, ACL reconstruction, Running, Landing, Cutting, Change of direction

## Abstract

**Purpose:**

Running, jumping/landing and cutting/change of direction (CoD) are critical components of return to sport (RTS) following anterior cruciate ligament reconstruction (ACLR), however the electromyographic (EMG) activity patterns of the operated leg during the execution of these tasks are not clear.

**Methods:**

A systematic review was conducted to retrieve EMG studies during running, jumping/landing and cutting/(CoD) in ACLR patients. MEDLINE, PubMed, SPORTDiscus and Web of Science databases were searched from 2000 to May, 2022 using a combination of keywords and their variations: “anterior cruciate ligament reconstruction” OR “ACLR”, “electromyography” OR “EMG”, “running”, “jumping” OR “landing”, “cutting” OR “change-of-direction” OR “CoD”. The search identified studies comparing EMG data during running, landing and cutting/(CoD) between the involved limb and contralateral or control limbs. Risk of bias was assessed and quantitative analyses using effect sizes were performed.

**Results:**

Thirty two studies met the inclusion criteria. Seventy five percent (24/32) of the studies reported altered EMG activity pattern of the ACLR leg during running, jumping/landing and cutting/(CoD) when compared with either the healthy control leg or the contra-lateral leg. Twelve studies showed decreased, delayed or earlier onset and delayed peak in quadriceps EMG activity with small to large effect sizes and 9 studies showed increased, delayed or earlier onset and delayed peak in hamstrings EMG activity with small to large effect sizes. Four studies showed a “hamstrings-dominant” strategy i.e. decreased quadriceps coupled with increased hamstrings EMG activity in both running and jumping/landing irrespective of graft type. One study reported that on the grounds of decreased quadriceps activity, lower hamstrings EMG activity was predictive of ipsilateral re-injury in ACLR patients.

**Conclusion:**

This systematic review of Level III evidence showed that the ACLR leg displays decreased quadriceps or increased hamstrings EMG activity or both despite RTS. Simultaneous decreased quadriceps and increased hamstrings EMG activity was shown for both running and jumping/landing. From a clinical perspective this “hamstrings dominant” strategy can serve as a protective mechanism against graft re-injury.

**Level of evidence:**

III.

**Supplementary Information:**

The online version contains supplementary material available at 10.1186/s40634-023-00603-1.

## Background

Anterior cruciate ligament (ACL) injuries most frequently occur during athletic activities/sports participation that involves some combination of running, jumping and cutting or changing direction (CoD) [59, 29, 76, 97, 104] and affect both amateur and professional athletes with injury rates ranging 3–15% per year [1, 50, 71]; though increasing trends in the average annual cases have been reported for both genders after adjusting for exposures [1, 95]. ACL reconstruction (ACLR) aims at reducing (though not eliminating) the risk for early-onset and accelerated progression of osteoarthritis (OA) [2, 65, 12–14, 20, 24, 63, 64, 77], propagate return to sport (RTS) to pre-injury levels [58] and eventually sustaining the same level of performance for the subsequent year [106].

Individuals who do RTS following ACLR, have an increased risk of re-injury [11, 75, 93, 108], with failure rates ranging ~ 3–5% in general population [37, 98] and ~ 5–17% in athletic populations [52, 56]. RTS is a complex, biopsychosocial process [5, 13, 17] that transcends choice of graft/graft-related functional outcomes [37, 75, 98], focuses on optimization of the functional recovery process [30, 31] and the restoration of “quality” of movement during running, jumping and cutting/ (CoD) [13, 31]. However, despite being cleared for RTS, ACLR athletes frequently display biomechanical alterations that are thought to predispose for either subsequent re-injury/graft failure or contra-lateral ACL injury [74, 35, 38, 60, 87, 103]. Whilst there is a host of factors impacting on these alterations [13, 17], neuromuscular activity patterns is a pivotal parameter because can be modified via training [68, 111].

Altered neuromuscular activity may be indicative of the ability to produce or accept force or identify potential areas of tissue overload [19, 41, 84]. Side to side differences in neuromuscular activity will result in altered movement quality, which in turn will induce further movement compensations and inappropriate patterns [13, 14, 19]. Finally it is not clear whether these differences in neuromuscular activity, are a function of time after surgery (thus reflecting tissue healing) [25, 47], functional criteria (highlighting restoration of motor control) [15, 16] or reflect pre-injury movement patterns [100, 110].

Thus, the objective of this systematic review was to synthesize the scientific literature regarding neuromuscular activity of the lower extremity muscles in adult, physically active ACL reconstructed patients during running, jumping and cutting/CoD tasks. The second aim was to examine whether EMG analyses of running, jumping and cutting/CoD could identify deficits with implications for either graft re-injury or contra-lateral ACL injury.

## Methods

The present systematic review was designed, conducted and analyzed according to the guidelines of Preferred Reporting of Items for Systematic Reviews and Meta-Analyses (PRISMA) [73] and followed the recommendations of the Cochrane group [44].

### Eligibility criteria

The following eligibility criteria had to be met in order for a study to be considered relevant for the purposes of the present systematic review

1. Study participants: male, female or both
2. Age ≥ 18 years
3. ACL injury that was treated surgically

Furthermore studies had to have used running or jumping or cutting/CoD as testing modality and apply EMG recordings of at least one lower extremity muscle. We narrowed our search to original, peer-reviewed article published in English. We did not set a limit to time since surgery to be used as an eligibility criterion. Participants’ activity level was not restricted to a particular level. Studies were eligible for inclusion if they used surface EMG to assess magnitude of activity (peak/mean muscle activity) or timing (onset/offset and duration) in the injured side of ACLR patients and compared it with a control group and/or the intact contra-lateral leg. Thus, we excluded studies on ACL deficient patients, studies where EMG recordings were used to derive model-driven muscle forces, studies comparing post- to pre-surgery neuromuscular activity or assessing muscle activity during walking, jogging, downhill or uphill running. Finally we excluded editorials, theses, book chapters and conference abstracts.

### Data sources

Our search was conducted from 2000 until May 2022 in the electronic databases MEDLINE/PubMed, SPORTDiscus and in the Web of Science. Furthermore, a manual search was done using the reference lists of included articles to identify additional and potentially relevant articles that had not been identified in the electronic searches.

### Search strategy and study selection

The literature search was undertaken to locate articles that evaluated lower extremity muscle activity during running, jumping/landing and cutting/CoD tasks in individuals having undergone ACL reconstruction. The keywords were: “anterior cruciate ligament”/ “ACL”, “reconstruction”/”ACL reconstruction”/”ACLR”, “EMG/ “electromyography”/neuromuscular activity”/”muscle activity”/”EMG amplitude”/”EMG timing”/”muscle on-set”/”pre-activity”/”re-activity”, “running”, “jumping”, “landing”, “change-of-direction”. Keywords were used individually and in various combinations with OR/AND operators as follows: (anterior cruciate ligament OR ACL) AND (reconstruction) OR (ACLR) AND (EMG OR Electromyography OR neuromuscular activity OR muscle activity OR EMG amplitude OR EMG timing OR muscle on-set OR pre-activity OR re-activity) AND (Running OR Jumping OR Landing OR cutting OR change-of-direction OR CoD). The search was performed by two authors and was further supplemented by manual search of the reference lists of papers selected from the initial database search. All titles and abstracts were independently screened by the two authors performing the search to identify potentially relevant papers based on eligibility criteria. The full manuscripts were subsequently retrieved and each paper independently assessed for inclusion/exclusion criteria by the same two authors. If their decision was not unanimous, a third reviewer assessed the eligibility of the article.

### Data collection process and data extraction

After final decision of all studies, data extraction for each included study was performed by two authors using a simple spreadsheet. The first author extracted study design, sample size and age of the ACLR and control group, graft type, time since surgery (in months) and activity level of groups. Furthermore the specific task per activity (running, jumping, cutting/CoD) was recorded along with EMG dependent variable and the muscle(s) studied. The EMG outcome of interest was registered as the reported comparison of the depended variable between ACLR leg and contra-lateral and/or control leg. Effect sizes (± 95%CI) were derived using sample size, mean and standard deviation of the reported values. When necessary data were unavailable, authors were contacted by email. The effect sizes were calculated according to the formula: Cohen d = mean (operated side) − mean (control or contra-lateral side)/SD (pooled) and were interpreted as small (≤ 0.4), moderate (≥ 0.5 up to 0.79) and large (≥ 0.8) [84].

### Risk of bias assessment

A modified version of the Downs and Black checklist [84, 94] was used to determine the risk of bias of all the included articles [32]. The checklist examines features related to i) reporting (objectives/hypotheses, main outcomes, characteristics of the participants, interventions, main confounders/findings, estimates of random variability, reporting of *p*-values), ii) external validity (subjects/staff/places/facilities), iii) internal validity (blinding subjects/assessors, data dredging, follow-up lengths/same time period between intervention and outcome for cases and controls, appropriateness of statistical tests/main outcome measures), iv) selection (patients and controls from same population and over same period of time, randomization, allocation concealed, adjustments for confounding, loss to follow-up) and v) power analysis. For the purposes of this systematic review, studies with a total score ≥ 17 were rated as being of a low risk of bias (thus of “high” methodological quality) [94]. Studies between 13–16 points were rated as being of “medium” quality, and studies ≤ 13 were rated as being of high risk of bias (thus of “low” methodological quality). No study was excluded due to low methodological quality; our aim was to synthesize all available data regarding neuromuscular activity. Two of the authors independently reviewed and scored all included studies based on the checklist (Supplementary Tables S1-S3). Any discrepancy was resolved in a consensus meeting, and a third reviewer was available if needed, but that was not required.

## Results

**Fig. 1:**
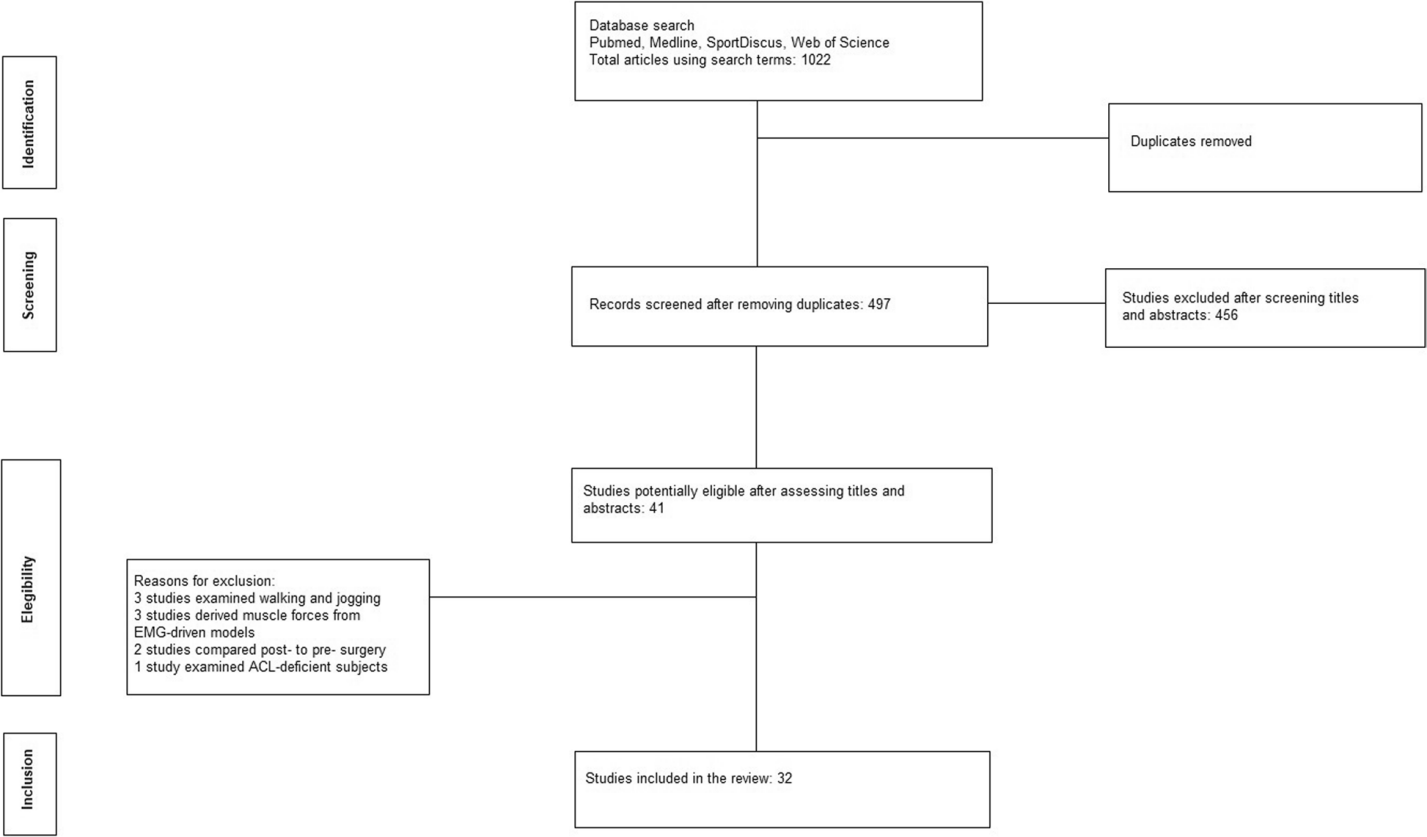
Flow chart of the search and included and excluded studies

Returning hits from the electronic database search and manual search were screened for duplicates. After applying inclusion and exclusion criteria according to PRISMA flowchart [73], a total of 32 studies (6 running studies, 22 jumping studies and 5 cutting/CoD studies, one study contributed to both jumping and cutting/CoD), involving 884 subjects -482 participants with ACLR and 402 healthy controls could be used for analysis. There were 49 ACLR participants and 47 controls in studies dealing with running, 356 ACLR participants and 251 controls in studies dealing with jumping and 77 ACLR participants and 65 controls in studies dealing with cutting/CoD. Studies were excluded mainly because EMG recordings were used to derive model-based muscular forces rather than muscle activity, participants did not receive reconstruction surgery or muscle activity of the reconstructed leg was compared to muscle activity at the pre-injury state. The flowchart describing the steps of the search is depicted in Fig. 1.

### Risk of bias assessment

Medium risk of bias was found for over 2/3 of the studies (22/32, 68.7%) [3, 4, 6, 7, 12, 21, 26–28, 34, 41, 46, 49, 61, 69, 80, 86, 90–92, 96, 101], 4/32 (12.5%) were of high methodological quality [10, 62, 78, 79] and 6/32 studies (18.8%) were of low quality [39, 72, 81, 82, 89, 109] (Supplementary Tables S1-S3). The main reasons for a medium to low methodological quality were due to unclear description of participants and/or prior interventions.

### Study design

Running studies were case–control [34, 46, 89, 91] or case series [90, 92] (Table 1). Jumping studies were case–control [3, 4, 7, 12, 21, 26, 28, 39, 49, 61, 69, 72, 80–82, 86, 96, 101] and case series [27, 41, 62, 78, 79] (Table 2). Cuttting/CoD studies were case–control [6, 10, 26, 82] or case-study [109] (Table 3). The case–control studies compared the ACLR participants with at least one control group (e.g. healthy controls), whilst the case series studies made a comparison between the ACLR and the intact contra-lateral leg.

**Table 1:** Participants characteristics for electromyographic running studies

**Study**	**ACLR-group**	**Control-group**	**Gender**	**Graft type**	**Post-op** **(months)**	**Activity level**
Einarsson et al., 2021 [34]	9 (27 ± 7.69 yrs) /12 (26 ± 3.84 yrs)	19 (35.4 ± 7.8 yrs)	M	BPTB (*n* = 9)/HS (*n* = 12)	7.0 ± 2.0	Participants in pivoting sports/runners
Jafarnezhadgero et al., 2021 [46]	14 (20.8 ± 0.3 yrs)	14 (21.3 ± 0.4 yrs)	M	HS (*n* = 14)	7.2 ± 1.1	Recreationally active (both groups)
Patras et al., 2012 [89]	14 (24.8 ± 5.3 yrs)	14 (21.7 ± 4.4 yrs)	M	BPTB (*n* = 14)	18.5 ± 4.3	Amateur soccer players (both groups)
Patras et al., 2011 [92]	14 (24.8 ± 5.3 yrs)	n/a	M	BPTB (*n* = 14)	18.5 ± 4.3	Amateur soccer players
Patras et al., 2010 [91]	14 (24.8 ± 5.3 yrs)	14 (21.7 ± 4.4 yrs)	M	BPTB (*n* = 14)	18.5 ± 4.3	Amateur soccer players (both groups)
Patras et al., 2009 [90]	9 (27.7 ± 3.5 yrs)	n/a	M	BPTB (*n* = 9)	19.2 ± 5.7	Amateur soccer players

Number in brackets corresponds to reference number; sample size and age in years (mean ± SD) are provided for ACLR and control group

*M* Male, *BPTB* Bone-patellar tendon-bone graft, *HS* Hamstrings graft, *post-op* time of testing since surgery in months (mean ± SD), *n/a* not available

**Table 2:** Participants characteristics for electromyographic jumping/landing studies

**Study**	**ACLR-group**	**Control-group**	**Gender**	**Graft type**	**Post-op** **(months)**	**Activity level**
Markström et al., 2022 [69]	10/11 (24.0 ± 3.9 yrs) ↑ fear 8/9(25.5 ± 5.8 yrs) ↓ fear	7/32 (22.4 ± 3.9 yrs)	M/F	HS (*n* = 38)	11.2 ± 2.0 ↑ fear 10.1 ± 2.0 ↓ fear	Active individuals (both groups)
He et al., 2022 [41]	30 (25.4 ± 1.1 yrs)	n/a	M	HS (*n* = 30)	9.9 ± 2.6	n/a
Alanazi et al., 2021 [4]	8/10 (26.1 ± 3.9 yrs)	8/10 (25.8 ± 3.5 yrs)	M/F	BPTB (*n* = 10)/HS (*n* = 8)	60.0 ± 18.0	Recreational soccer players (both groups)
Behnke et al., 2021 [7]	5/4 (33.4 ± 10.5 yrs)	6/3 (38.6 ± 5.9 yrs)	M/F	BPTB (*n* = 2)/HS (*n* = 7)	145.3 ± 15.2	n/a
Rocchi et al., 2020 [96]	15 ( 21 ± 3 yrs)/11 ( 21 ± 5 yrs)	15 (21.7 ± 4.4 yrs)	M	BPTB (*n* = 15)/HS (*n* = 11)	6.0 ± 1.2	Competitive sports participants
Alanazi et al., 2020 [3]	8/10 (26.1 ± 3.9 yrs)	8/10 (25.8 ± 3.5 yrs)	M/F	BPTB (*n* = 10)/HS (*n* = 8)	60.0 ± 18.0	Recreational soccer players (both groups)
Burland et al., 2020 [21]	10/16 (20.2 ± 2.7 yrs)	4/4 (23.3 ± 1.8 yrs)	M/F	BPTB (*n* = 21)/HS (*n* = 5)	26 ± 20	n/a
Smeets et al., 2020 [101]	15/6 (23.8 ± 4.2 yrs)	15/6 (21.5 ± 1.5 yrs)	M	BPTB (*n* = 14)	8.6 ± 2.0	Participants in pivoting sports (both groups)
Dashti Rostami et al., 2020 [27]	20 (26.77 ± 3.75 yrs)	n/a	M	BPTB (*n* = 9)/HS (*n* = 7)/allograft (*n* = 4)	26.55 ± 4.31	Participants in pivoting sports
Dashti Rostami et al., 2019 [28]	12 (23.8 ± 5.5 yrs)	12 (24.9 ± 2.1 yrs)	M	BPTB (*n* = 6)/HS (*n* = 4)/allograft (*n* = 2)	23.8 ± 6.3	Participants in pivoting sports
Palmieri-Smith et al., 2019 [86]	5/2 (17.1 ± 2.7 yrs)	5/2 (22.6 ± 3.3 yrs)	M/F	BPTB (*n* = 7)	7.6 ± 2.0	n/a
Lessi et al., 2018 [62]	7 ( 24 ± 2.8 yrs)/7 (24.7 ± 5.3 yrs)	n/a	M/F	BPTB (*n* = 5)/HS (*n* = 9)	21.1 ± 6.8 (M) 24.2 ± 9.5 (F)	Recreational athletes
Jordan et al., 2017 [49]	6 (26.5 ± 5.8 yrs)/5 (23.6 ± 1.8 yrs)	6 ( 23.3 ± 3.3 yrs)/5 (21.8 ± 3.2 yrs)	M/F	BPTB (*n* = 1)/HS (*n* = 7)/allograft (*n* = 3)	36 ± 24	International level skiers
Lessi et al., 2017 [61]	13/7 (23.6 ± 2.9 yrs)	13/7 (25.1 ± 4.2 yrs)	M/F	BPTB (*n* = 5)/HS (*n* = 9)	> 12	Recreational athletes
Melińska et al., 2015 [72]	6 (26.2 ± 2.3 yrs)	22 (25.1 ± 4.3 yrs)	M	n/a	~ 8	n/a
Nyland et al., 2014 [79]	32/33 (26.2 ± 2.3 yrs)	n/a	M/F	BPTB (*n* = 25)/HS (*n* = 7)/other (*n* = 33)	60 ± 30	Participants in pivoting sports
Ortiz et al., 2014 [80]	14 (28.5 ± 4.6 yrs)	16 (27.7 ± 3.9 yrs)	F	HS (*n* = 14)	n/a	Collegiate volleyball players/ Collegiate players in pivoting sports
Ortiz et al., 2011 [82]	14 (25.4 ± 3.1 yrs)	14 (24.5 ± 2.6 yrs)	F	BPTB (*n* = 9)/HS (*n* = 2)/other (*n* = 2)	84 ± 40	Recreational athletes
Nyland et al., 2010 [78]	35/35 (26.2 ± 2.3 yrs)	n/a	M/F	BPTB (*n* = 25)/HS (*n* = 7)/other (*n* = 38)	60 ± 30	Participants in pivoting sports
Gokeler et al., 2010 [39]	6/3 (28.4 ± 9.7 yrs)	8/3 (26.3 ± 5.5 yrs)	M/F	BPTB (*n* = 9)	4 ± 0.2	Participants in pivoting sports
Bryant et al., 2009 [12]	15 (30.9 ± 7.3 yrs)/13 (22.9 ± 3.8 yrs)	22 (29.0 ± 8.2 yrs)	M	BPTB (*n* = 14)/HS (*n* = 13)	15.1 ± 5.0/14.2 ± 4.5	Recreational athletes
Ortiz et al., 2008 [81]	14 (25.4 ± 3.1 yrs)	14 (24.5 ± 2.6 yrs)	F	BPTB (*n* = 9)/HS (*n* = 2)/other (*n* = 2)	84 ± 40	Recreational athletes

Number in brackets corresponds to reference number; sample size and age in years (mean ± SD) are provided for ACLR and control group, depending on study age is provided separately for males and females or as one sample

*↑ fear* high kinesiophobia ACLR-group, *↓ fear* low kinesiophobia ACLR-group, *M* Male, *F* Female, *BPTB* Bone-patellar tendon-bone graft, *HS* Hamstrings graft, *allograft* synthetic graft, *other* autograft other than BPTB or HS, *post-op* time of testing since surgery in months (mean ± SD), *n/a* not available

**Table 3:** Participants characteristics for electromyographic cutting/CoD studies

**Study**	**ACLR**	**Control-group**	**Gender**	**Graft type**	**Post-op** **(months)**	**Activity level**
Arumugam & Hager, 2022 [6]	9/25 (25.8 ± 4.2 yrs)	3/19 (26.3 ± 5.5 yrs)	M/F	BPTB (*n* = 7)/HS (*n* = 3)	33.7 ± 32	n/a/National level athletes in team sports
Zebis et al., 2017 [109]	1	n/a	F	HS (*n* = 1)	12	Elite soccer player
Briem et al., 2016 [10]	18 (22.7 ± 3.5 yrs)	18 (21.5 ± 2.7 yrs)	F	HS (*n* = 18)	12–72	National level handball, basketball, football players (both groups)
Coats-Thomas et al., 2013 [26]	4/6 (25.8 ± 4.2 yrs)	8/3 (26.3 ± 5.5 yrs)	M/F	BPTB (*n* = 7)/HS (*n* = 3)	≥ 60	n/a
Ortiz et al., 2011 [82]	14 (25.4 ± 3.1 yrs)	14 (24.5 ± 2.6 yrs)	F	BPTB (*n* = 9)/HS (*n* = 2)/other (*n* = 2)	84 ± 40	Recreational athletes

Number in brackets corresponds to reference number; sample size and age in years (mean ± SD) are provided for ACLR and control group

*M* Male, *F* Female, *BPTB* Bone-patellar tendon-bone graft, *HS* Hamstrings graft, *other* autograft other than BPTB or HS, *post-op* time of testing since surgery in months (mean ± SD), *n/a* not available

### Participants

The sample size for the ACLR participants, ranged from *n* = 1 [109] to a maximum of *n* = 65 [78]. Running studies recruited exclusively males [34, 46, 89–92] receiving the median or medial 1/3 of the bone-patella tendon-bone (BPTB) [89–92], hamstrings HS [46] or a mixed BPTB and (HS) [34] grafts and their activity level was mainly amateur soccer players [89–92] (Table 1). Jumping studies recruited exclusively males [12, 27, 28, 41, 72, 96, 101], females [80–82] or both males and females [3, 4, 7, 21, 39, 49, 61, 62, 69, 78, 79, 86] receiving BPTB [39, 86, 101], hamstrings HS [41, 69, 80] or mixed BPTB and (HS) [3, 4, 7, 12, 21, 27, 28, 49, 61, 62, 78, 79, 81, 82, 96] grafts and included mostly some form of active sport-participants (Table 2). Cutting/CoD studies recruited exclusively females [10, 82, 109] or both males and females and their activity level was registered mainly as national level team-sport athletes (Table 3). Time since surgery ranged from as low as 4–8 months [34, 39, 46, 72, 86, 96, 101] to ≥ 60 [3, 4, 7, 26, 78, 79, 81, 82].

### Interventions

The number of muscles assessed ranged 1–9 and investigators mainly assessed muscle activity around the thigh and recorded EMG signal from rectus femoris (RF), vastus lateralis (VL), vastus medialis (VM), biceps femoris (BF) and semitendinosus/semimebranosus (ST/SM) (Tables 4, 5 and 6). Less often EMG signal from gluteus maximus/medius (GMAX/GMED) and calf muscles such medial and lateral gastrocnemius (GM/GL) and soleus (SO) was also recorded (Tables 4, 5 and 6).

**Table 4:** Intervention, EMG variable, muscle(s) tested and main outcome for running

**Study**	**Task**	**EMG variable**	**Muscles**	**EMG outcome of interest for ACLR vs. intact/control leg**	**ES**
Einarsson et al., 2021 [34]	Treadmill running at 12, 14 and 16 km·h^−1^	Peak EMG amplitude	BF, ST	↓ SM/ST EMG activity for hamstring graft	0.67(0.01;1.33)
Jafarnezhadgero et al., 2021 [46]	Running at 12 km·h^−1^ with 3 running strike patterns	Peak EMG amplitude	RF, VL, VM, BF, ST, GMD, GM, TA	↓ EMG activity for VM and ↑ for BF in ACLR (early stance) /↓ EMG activity for VL, VM, TA, GM in ACLR (late stance)	0.90–1.71 (VM), 0.83 (VL), 0.85 (BF), 0.94 (TA), 1.71 (GM)
Patras et al., 2012 [89]	10 min treadmill running at ~ 65% VO_2_max/10 min treadmill running at ~ 85% VO_2_max	Peak EMG amplitude during stance	VL, BF	Lack of progressive ↑ in EMG activity for VL in ACLR during 85% VO_2_max Progressive ↑ in EMG activity for BF in ACLR during 85% VO_2_max	0.85(0.25;1.39) (VL, intact), 1.02(0.27;1.77) (VL, control), 0.28(0.09;0.49) (BF, intact), 0.33(0.12;0.45) (BF, control),
Patras et al., 2011 [92]	10 min treadmill running at ~ 85% VO_2_max	Peak EMG amplitude during stance	VL	↓ correlation between EMG activity and lactate threshold for ACLR	n/a
Patras et al., 2010 [91]	10 min treadmill running at ~ 65% VO_2_max/10 min treadmill running at ~ 85% VO_2_max	Peak EMG amplitude during stance	VL	Lack of progressive ↑ in EMG activity for VL in ACLR during 85% VO_2_max	0.57(0.14;0.99) (intact), 0.42(0.08;0.77) (control)
Patras et al., 2009 [90]	10 min treadmill running at ~ 65% VO_2_max/10 min treadmill running at ~ 85% VO_2_max	Peak EMG amplitude during stance	VL	Lack of progressive ↑ in EMG activity for VL in ACLR during 85% VO_2_max	0.69(0.34;1.04) (intact), 0.58(0.22;0.93) (control)

Number in brackets corresponds to reference number

*VO*_*2*_*max* maximal oxygen uptake, *EMG* Electromyographic, *BF* Biceps femoris, *ST* Semitendinosus, *RF* Rectus femoris, *VL* Vastus lateralis, *VM* Vastus medialis, *GMD* Gluteus medius, *GM* Gastrocnemius medial, *TA* Tibialis anterior, *↑* increase, *↓* decrease, *ES* Effect size (Cohen’s d) reported as mean(95%CI), *n/a* not available

**Table 5:** Intervention, EMG variable, muscle(s) tested and main outcome for jumping/landing

**Study**	**Task**	**EMG variable**		**Muscles**	**EMG outcome of interest for ACLR vs. intact/control leg**	**ES**
Markström et al., 2022 [69]	Single-leg side-hop	Normalized peak EMG amplitude		BF, ST, VL, VM	↑ EMG activity for BF of high-fear (post-impact)	1.20(0.61;1.76) (control), 1.42(0.68;2.10) (low-fear)
He et al., 2022 [41]	Single-leg forward hop	Normalized (%MVC) EMG amplitude		BF, SM, VL, VM	Delayed EMG activity for VM (onset)	0.59(0.06;1.09) (control)
Alanazi et al., 2021 [4]	Double-leg forward jump before and after a Wingate test	Normalized mean EMG amplitude		RF, VL, VM, BF, ST, GM	No diff in EMG activity	n/a
Behnke et al., 2021 [7]	Single-leg forward hop	Normalized EMG amplitude		RF, VM, BF, ST, GM, GL	Shorter EMG activity onset for BF	0.87(-0.14;1.79)
Rocchi et al., 2020 [96]	Single-leg forward jump	Normalized (%MVC) EMG amplitude		RF, VL, VM, BF, ST	↑ EMG activity duration (pre-impact)	1.59(0.73;2.36) (quadriceps), 1.49(0.64;2.25) (hamstrings)
Alanazi et al., 2020 [3]	Planned and unplanned double-leg forward jump	Normalized mean EMG amplitude		RF, VL, VM, BF, ST, GM	↓ EMG activity for GM (post-impact)	0.78(0.08;1.44) (planned), 0.95(0.24;1.61) (unplanned)
Burland et al., 2020 [21]	Single-leg forward hop	Normalized peak EMG amplitude		VL	↓ EMG activity for VL	0.90(0.06;1.70) (control)
Smeets et al., 2020 [101]	Single-leg forward hop, single-leg side-hop, single-leg vertical hop with 90° rotation	Normalized (%MVC) EMG amplitude		VL, VM, BF, ST, GM, GL, GMED	No diff in EMG activity	n/a
Dashti Rostami et al., 2020 [27]	Single-leg drop jump	Normalized (%MVC) EMG amplitude		VL, VM, BF, ST, GM, GL	No comparative leg	n/a
Dashti Rostami et al., 2019 [28]	Single-leg drop jump	Normalized (%MVC) EMG amplitude		GMED, AL	↓ EMG activity for GMED (post-impact)	0.75(-0.10;1.55)
Palmieri-Smith et al., 2019 [86]	Single-leg forward hop	Normalized peak EMG amplitude		VL, BF, GL	↓ EMG activity for VL (post-impact)	1.86(0.61;3.11)
Lessi et al., 2018 [62]	Single-leg drop jump before and after a fatigue protocol	Normalized mean EMG amplitude		VL, GMED, GMAX	Lack of progressive ↑ in EMG activity for VL post-fatigue (post-impact)	0.79
Jordan et al., 2017 [49]	80 s repeated squat jumps	Normalized (%MVC) EMG amplitude		VL, VM, BF, ST	↓ EMG activity (VL + VM) (pre-impact/post-impact) ↑ EMG activity (BF + ST) (pre-impact/post-impact)	Q pre: 2.62(1.48;3.76)-1.86(0.86;2.86), Q post: 2.17(1.12;3.23)-1.82(0.83;2.81) H pre: 2.30(1.22;3.37)-1.44(0.46;2.32) H post: 2.90(1.70;4.09)-0.93(0.05;1.81)
Lessi et al., 2017 [61]	Single-leg drop jump before and after a fatigue protocol	Normalized EMG amplitude		VL, BF, GMAX, GMED	No difference in EMG activity	n/a
Melińska et al., 2015 [72]	Double-leg drop-jump	Normalized EMG amplitude		VM, ST, GM, GL	↑ EMG activity for GM, GL (post-impact)	n/a
Nyland et al., 2014 [79]	Single-leg forward hop	Normalized (%MVC) EMG amplitude		GMAX, VM, ST, GM	↑ EMG activity for GMAX, GM for high vs. low “performance” group (pre-impact/post-impact)	GMAX: 0.69(0.05;1.33), GM: 0.64(0.01;1.28)
Ortiz et al., 2014 [80]	Double-leg drop jump Single-leg drop jump	Normalized EMG amplitude		RF, VL, VM, BF, ST	↓ Quadriceps and hamstrings EMG activity (double-leg drop jump)/ ↑ EMG activity quadriceps (single-leg drop jump)	Quadriceps: 0.22, Hamstrings:0.18/ Quadriceps: 0.17
Ortiz et al., 2011 [82]	Single-leg side hop	Normalized EMG amplitude		GMAX, RF, BF, ST	No diff in EMG activity	n/a
Nyland et al., 2010 [78]	Single-leg CMJ	Normalized (%MVC) EMG amplitude		GMAX, VM, ST, GM	↑ EMG activity for GMAX, (pre-impact/post-impact) ↑ EMG activity for GM (male only) (post-impact)	GMAX: 0.54(0.06;1.01) (M), 0.73(0.24;1.21) (F), GM: 0.60(0.12;1.08) (M)
Gokeler et al., 2010 [39]	Single-leg forward hop	Non-normalized EMG		GMAX, BF, ST, RF, VL, VM, GM, GL, SO	Earlier EMG onset for GMAX, BF, RF, GM, GL, SO	VM:1.19, RF: 1.22, SM: 1.09, GMAX:1.62, MG:1.07, LG: 1.62, SO: 2.53
Bryant et al., 2009 [12]	Double-leg forward hop	Non-normalized EMG		VL, VM, BF, ST	No difference in EMG activity (onset)	n/a
Ortiz et al., 2008 [81]	Single-leg drop jump Single-leg box jump		Normalized EMG amplitude	GMAX, BF, ST, RF	↑ EMG activity for GMAX, RF (single-leg drop jump)	GMAX:0.29, RF:0.36

Number in brackets corresponds to reference number

*CMJ* Counter movement jump, *EMG* Electromyographic, *%MVC* % Maximum voluntary contraction, *BF* Biceps femoris, *ST* Semitendinosus, *RF* Rectus femoris, *VL* Vastus lateralis, *VM* Vastus medialis, *GMD* Gluteus medius, *GM* Gastrocnemius medial, *GL* Gastrocnemius lateral, *TA* Tibialis anterior, *SM* Semimembranosus, *GMAX* Gluteus maximus, *SO* Soleus, *pre-impact* prior to ground contact, *post-impact* post ground contact, *↑* increase, *↓* decrease, *ES* Effect size (Cohen’s d) reported as mean(95%CI), *Q* Quadriceps, *H* Hamstrings, *n/a* not available

**Table 6:** Intervention, EMG variable, muscle(s) tested and main outcome for change of cutting/CoD

**Study**	**Task**	**EMG variable**	**Muscles**	**EMG outcome of interest for ACLR vs. intact/control leg**	**ES**
Arumugam & Hager, 2022 [6]	Single-leg forward-hop + unanticipated single-leg diagonal-hop	Normalized peak EMG amplitude	BF, ST, VL, VM	No diff in EMG activity (as Q/H ratio)	n/a
Zebis et al., 2017 [109]	Side-cutting hop	Normalized EMG amplitude	BF, SM, VL	↓ EMG activity for ST (pre-impact)	n/a
Briem et al., 2016 [10]	Cross-over triple hop	Normalized (%MVC) EMG amplitude	BF, ST	↓ EMG activity for BF/ ↑ EMG activity for ST	n/a
Coats-Thomas et al., 2013 [26]	Single-leg forward-hop + unanticipated diagonal cut	Normalized EMG amplitude	RF, VM, BF, ST, GM, GL	Delayed peak EMG timing for RF, VM, BF, GM	RF:1.45 (M)-0.48 (F), VM:1.48 (M)-0.56 (F), BF:1.91 (M)-0.39 (F), GM:1.28 (M)-0.85 (F)
Ortiz et al., 2011 [82]	Cross-over hop	Normalized EMG amplitude	GMAX, RF, BF, ST	No difference in EMG activity	n/a

Number in brackets corresponds to reference number

*EMG* Electromyographic, *%MVC* % Maximum voluntary contraction, *BF* Biceps femoris, *ST* Semitendinosus, *RF* Rectus femoris, *VL* Vastus lateralis, *VM* Vastus medialis, *GM* Gastrocnemius medial, *GM* Gastrocnemius lateral, *Q/H ratio* Quadriceps to hamstrings ration,*↑* increase, *↓* decrease, *ES* Effect size (Cohen’s d) reported as mean, *M* Male, *F* Female, *n/a* not available

Running tasks involved conventional typically treadmill [34, 89–92] but also over-ground [46] running was used (Table 4). Jumping tasks included hops (single- and double- leg) [7, 21, 39, 41, 69, 79, 82, 86, 101], jumps [3, 4], drop jumps (single- and double- leg) [27, 28, 61, 62, 72, 80, 81], countermovement jumps (single-leg) [78] and box jumps (single-leg) [81] (Table 5). Finally cutting/CoD tasks involved a combination of hops interspersed with a change in direction such as cross-over hops [10, 82], or forward hop followed by diagonal hop [6, 26] (Table 6). In addition, some studies even investigated the influence of fatigue on neuromuscular activity during either running [89–92] or jumping tasks [4, 49, 61, 62].

### Outcomes

All included studies assessed muscle activity using surface EMG according to standardized procedures and guidelines provided by the SENIAM project (Surface Electromyography for the Non-Invasive Assessment of Muscles) [42]. The EMG-related dependent variables included peak and/or mean amplitude, timing of peak muscle activity, preparatory and reactive muscle activity and onset of muscle activation. The outcome variables were expressed either as a percentage of maximum voluntary (isometric) contraction (%MVC), as a percentage of peak muscle activity during the task or as microvolts/milliseconds. Seventy five percent of the included studies reported statistically significant differences in muscle activity patterns during running, jumping and cutting/CoD tasks when the ACLR leg was compared to the contra-lateral intact and/or control leg [3, 7, 10, 21, 26, 28, 34, 39, 41, 46, 49, 62, 69, 72, 78–80, 86, 89–92, 96, 109] (Tables 4, 5 and 6).

Quadriceps muscles showed decreased EMG amplitude during running [46, 89–91] (Table 4) and jumping [21, 49, 62, 80, 86] (Table 5), increased EMG amplitude during jumping [80, 81, 96] (Table 5), earlier [39] or delayed [41] EMG onset during jumping (Table 5), delayed [26] EMG peak during cutting or no difference in EMG amplitude or onset during jumping [3, 4, 7, 12, 61, 69, 82] or cutting [6, 82, 109] (Tables 5 and 6]. Hamstrings muscles showed increased [46, 89] or decreased [34] EMG amplitude during running (Table 4), increased [49, 69] or decreased [80] EMG amplitude, earlier onset or delayed peak [39, 69, 96], or no difference [41, 61, 78, 79, 81, 82] during jumping (Table 5) and increased [10, 109] or decreased [10] EMG amplitude, delayed peak [26] or no difference [6, 82] during cutting (Table 6). Additionally GM/GL showed decreased EMG amplitude during running [46] (Table 4), increased [72, 78, 79], decreased [3] or no difference [4, 7, 86, 101] in EMG amplitude during jumping (Table 5) and delayed peak in EMG amplitude during cutting/CoD [26] (Table 6). Finally, GMAX/GMED showed increased [39, 78, 79, 81] or decreased [28] EMG amplitude during jumping and no difference in EMG amplitude during running [46] (Table 4) and jumping [46, 61, 62, 82, 101] (Table 5).

Research exclusively on males showed decreased or delayed quadriceps EMG activity in four studies involving running [34, 46, 89–91] (Table 4) and decreased [41], increased [96] or no difference [12, 101] during various hops (Table 5). Studies on males report increased BF [46, 89] or decreased [34] ST/SM EMG activity (specifically in individuals operated with hamstrings graft) during running or no difference during various jumps [12, 41, 96, 101] (Table 5). Decreased EMG activity during jumping in males has been reported for GM [41], and GMED [28]. In females increased [80, 81], decreased [80] or no difference [82] in quadriceps activity has been reported during various jump protocols (Table 5). In addition females have decreased BF EMG activity during jumping [80] and cutting/CoD [10]; others however report no difference [81, 82] (Table 5). Studies with both male and female participants report decreased quadriceps activity, delayed peak or earlier onset [21, 26, 39, 49, 62, 86] but others report no difference [4, 7, 61, 69, 78, 79] during jumping. Hamstrings display increased activity, delayed peak or earlier onset [7, 26, 39, 49, 69], whilst no difference is reported in others [4, 61, 78, 79] during jumping. Those who report no differences in either quadriceps or hamstrings EMG activity, do report increased GMAX activity [78, 79].

Decreased quadriceps activity or earlier onset following ACLR with BPTB graft is reported in all [39, 86, 89–92] but one [101] studies. Increased hamstrings activity is reported during running [89]. Regarding ACLR with hamstrings graft decreased quadriceps activity or onset has been reported [41, 46, 80] but no difference has also been reported [69, 109]. Regarding hamstrings muscle activity in these patients, increased [46, 49, 80] and decreased BF [10] has been reported. In addition ST/SM activity showed no difference [46] or decreased activity [109] in patients receiving hamstrings graft. Studies with mixed sample of grafts report decrease in quadriceps activity [49, 62], increase in quadriceps activity [81] or duration [96] or no difference [3, 4, 7, 12, 61, 78, 79, 82]. Regarding hamstrings, activity is decreased and there is also shorter BF onset [7, 34, 49] but other report no difference in hamstrings activity [3, 4, 12, 61, 82].

## Discussion

The aim of the present review was to synthesize the scientific literature regarding EMG activity of the lower extremity muscles in adult, physically active ACLR individuals during running, jumping/landing and cutting/CoD tasks. All three tasks are important elements in the rehabilitation process of ACLR participants aiming to RTS [15, 16, 84] and thus any EMG alterations are deemed high relevant.

Results on running show a decrease in muscle activity of the VM (early stance) and VL, VM (late stance) with moderate to large effect sizes [46] as well as reduced progressive recruitment of the VL during late stance with mainly moderate effect sizes [89–91]. In addition, increased BF EMG activity (large effect size) [46] as well as increased progressive recruitment of the BF during the stance phase (small effect size) have been reported [89]. The coupled reduction in VM/VL and increase in BF activity muscle activity has been reported for both HS [46] and BPTD graft [89–91]. In non-injured subjects the preferential increase in agonist EMG activity, which characterizes the “quadriceps-dominant” strategy, is considered to reflect the physiological response to the accumulation of metabolic fatigue [22, 48, 66, 89, 113] as well as a biomechanical consequence that is associated with better neuromuscular control of the joint during fatigue [53, 54, 83]. Thus, following reconstruction these studies suggest a replacement of the typical “quadriceps-dominant” strategy by a “hamstrings-dominant” strategy for the ACLR leg during running, aiming to dynamically stabilize the reconstructed limb and decrease the anterior stress applied to the ACL graft [46, 89]. Importantly the lack of the anticipated increase in agonist EMG activity has also been reported for the VL muscle during single-leg drop-jump before and after a fatigue protocol [62]. Therefore the “hamstrings-dominant” strategy may reflect either an alteration of the local physiological response to accumulating fatigue or a biomechanical adaptation to stabilize the joint under fatigue. This hypothesis is further supported by evidence showing that aerobic endurance is more strongly correlated to the relative increase in VL EMG activity on the intact contra-lateral leg compared to the corresponding increase in the EMG activity of the ACLR leg during high-intensity fatiguing running [91]. From a clinical perspective the establishment of a “hamstrings-dominant” strategy during high-intensity running offers a potential protective mechanism after a unilateral ACLR. In addition it has been shown that BPTB ACLR individuals who experienced a secondary ipsilateral ACL injury have lower hamstrings activity compared to BPTB ACLR individuals who did not [86]. Thus, the lack of high BF activity following ACLR may increase the risk for ipsilateral ACL injuries at least in BPTD grafts [86] or increase the chances for a hamstring injury [105] irrespective of graft type [34].

Jumping studies are the most abundant, possible because of the clinical relevance of the hopping tests [56]. Results regarding timing of muscle activity are mixed with some studies reporting earlier onset (large effect sizes for all tested muscles) [7, 39] or longer duration during pre-impact (large effect sizes for both quadriceps and hamstrings) [96], but others have reported no significant difference in muscle onset [12] or even a delayed muscle onset (moderate effect size for VM only) [41]. Thus, three studies with large effect sizes show earlier onset or the longer duration at pre-impact for both quadriceps and hamstrings which may indicate increased pre-tension that serves as a protective mechanism by stiffening the joint for the subsequent impact [33]. A line of criticism is that the patients in these two studies were examined ~ 4–6 months following surgery; the corresponding time since surgery for the other studies was ~ 15 months [12] and ~ 60 months [41]; thus it is possible that the early EMG onset is observed only in the initial rehabilitation period. In addition the apparently opposite in direction trends regarding the onset of VM activity [39, 41] may be attributed to the different methodology. Indeed Gokeler et al., (2010) [39] defined the muscle onset as the first muscle burst in EMG activity before landing, whereas He et al., (2022) [41] defined the onset as the rising of linear envelopes representing muscle burst.

EMG amplitude has been examined both pre-impact (preparatory muscle activity) [28, 49, 86] and post-impact following initial contact (reactive muscle activity) [3, 21, 28, 49, 62, 69, 72, 78–81, 86]. Five studies indicate decreased post-impact activity for quadriceps (small to large effect sizes) [21, 49, 62, 80, 86], whilst three studies suggest increased post-impact activity for the quadriceps (small effect sizes) [72, 80, 81]. Regarding hamstrings, two studies report increased activity post-impact (large effect sizes) [49, 69] and one study reported decreased hamstrings activity (small effect size) [80]. Furthermore data for muscles above or below the knee show increased activity for GMAX (small to moderate effect sizes) [78, 79, 81] or decreased GMED activity (moderate effect size) [28] and either increased (moderate effect sizes) [72, 78, 79] or decreased (large effect size) [3] GM activity. The decreased quadriceps post-impact activity may bear resemblance to the “quadriceps-avoidance” gait that has been observed during hop landing in ACL-deficient subjects [8, 36]; however others indicate increased quadriceps post-impact activity [72, 80, 81] or no change in quadriceps activity [4, 12, 61, 82, 101]. In fact the same subjects show decreased quadriceps activity during double-leg drop jump and increased quadriceps activity during single-leg drop jump [80], therefore depending on the task the decreased quadriceps activity may represent merely a variation in landing strategy with subjects having increased use of the non-operate leg to control landing. Thus, from a clinical perspective regarding landing tasks most studies point towards reduced quadriceps activity which may be potentially dangerous, since decreased quadriceps activity is thought to lead to re-injury and may contribute to the development of post-traumatic OA [107, 85] and increased hamstrings activity that may act as a protector against anterior tibial shear [83]; however increased hamstring activity may also be present in ACLR patients with high degree of kinesiophobia [69]. The simultaneous decreased quadriceps activation coupled with increased hamstrings activation that characterizes the “hamstring-dominant” strategy reported in running [46, 89] appears to be also present in landing tasks [49, 86]. In fact in the latter study it was reported that lower hamstrings activity in ACLR subjects was predictive of subsequent re-injury [86]. Furthermore a recurring pattern that deserves further notice is the increased activation of the GMAX post-impact, which has been described as “hip-bias” compensation [78, 79, 81] and has been observed at the involved lower extremity among most subjects who report high perceived sports capability compared to pre-injury status [78, 79]. The authors hypothesized that these compensations may be related to a neuro-sensory deficit and subsequent CNS sensorimotor re-organization [79].

Regarding cutting/CoD in a limited number of studies, cross-over hops have been associated with either decreased or no change in BF [10, 82], whilst hop followed by unanticipated diagonal hop ort cut showed either no difference or delayed EMG activity of the quadriceps and hamstrings activity (with small to large effect sizes) [6, 26]. The delayed EMG activity seen in the ACLR leg during an unanticipated cut was considered to reflect sensory deficit in the operated knee [26], whilst the increased medial hamstring activity coupled with decreased lateral hamstring activity was viewed as a potential injury mechanism for the contra-lateral leg [10]. These limited studies exhibit inherent limitations that do not allow application as a monitoring tool during rehabilitation. CoD in chaotic sports environments is influenced by a multitude of factors and cannot be simply simulated as a series of hops [70].

Collectively we examined neuromuscular activity patterns regarding lower extremity muscles in individuals with primary ACLR during common athletic tasks such as running, landing and CoD/cutting. We observed reduced quadriceps coupled with increased hamstrings activity (i.e. a “hamstrings-dominant” strategy) for the operated leg during running and landing irrespective of graft [46, 49, 89] which may offer a plausible explanation for contra-lateral ACL injuries. In addition, BPTB ACLR individuals who experienced a secondary ipsilateral ACL injury had lower hamstrings activity during a landing task compared to BPTB ACLR patients who did not [86]; thus further supporting the importance of high hamstrings activity following ACLR given that reduced quadriceps activity of the operated leg has been established for running [90, 91] and jumping/landing [21, 49, 62, 80, 86]. Increased hamstrings activity may also indicate kinesiophobia especially if coupled with reduced functional performance [69]; on the contrary high performing sub-groups of patients may demonstrate higher GMAX activity [78, 79, 81]. A possibility for lower (medial) hamstrings activity may appear in ACLR with a hamstrings graft [34]. The reported neuromuscular alterations were observed despite that ACLR participants had completed all clinical criteria (deficit on isokinetic and functional field testing, pain-free, no swelling on swipe test and full ROM allowing resumption of high speed running) and had even RTS which may underscore the need for prolonged movement re-training [13, 15–17], interventions to modulate neuromuscular activity as soon as possible with minimal burden on joint/graft loading [40, 102] as well as targeting other strength-related qualities such as rate of force development [18].

Neuromuscular activity during athletic maneuvers in healthy subjects showed some potential in identifying individuals at increased risk for suffering ACL injury [100, 110]. Following RTS after primary ACLR there is the risk for ipsilateral re-injury or contra-lateral ACL injury [51, 55, 57, 67, 88, 93, 98]. Therefore examining EMG activity patterns during athletic tasks following primary ACLR appears to be an important factor to consider in establishing a potential connection between time since surgery, RTS and risk for re-injury. The risk for contra-lateral ACL injury following primary BPTB ACLR is higher compared to the risk of ipsilateral re-injury [51, 67, 88, 112], whilst primary HS ACLR is associated with higher rate of graft failure compared to contra-lateral ACL injury [51, 55, 57, 67, 88, 93, 98]. In addition at 15 years of follow up contra-lateral ACL tears are significantly more likely than graft failures [67], but graft failure is higher in hamstrings ACLR compared to BPTB ACLR [112]. Our results indicate that decreased quadriceps activity following ACLR is rather graft independent and has been reported for either BPTB [39, 86, 89–92] or HS [41, 46, 80] grafts. Whilst the down-regulation of quadriceps activity may be straightforward in the case of BPTB ACLR [19], the reported hamstrings neuromuscular facilitation that occurs even in the case low/medium hamstrings strength deficit, although “protective” in nature (by theoretically reducing shear forces at the knee joint), may be responsible for this down-regulation of quadriceps activation through reciprocal inhibition [85] regardless of graft selection. Thus optimization of quadriceps muscle function is always a high priority [19]; therefore we consider pivotal the role of hamstrings in the RTS following ACLR [14, 99]. Given that graft failure may occur when the hamstrings are actively lengthening to resist anterior tibial translation [9] and that hamstrings muscle tears mainly occur when the hamstrings act eccentrically to brake the knee extension at the end of the swing phase during running ([23], eccentric strengthening of the hamstrings following ACLR seems highly relevant; in fact persistent flexor strength deficits may not be revealed by “gold standard” isokinetic concentric testing, but with more functional eccentric strength testing [45]. In addition given that hamstrings act as both knee flexor and hip extensor, balancing “knee-dominant” and “hip-dominant” exercises may result in optimal functioning of the hamstrings especially during high-intensity running when their hip moment arm is double their knee moment arm [43]. Contra-lateral secondary ACL injuries following ACLR are more common compared to graft re-injuries [51, 67, 88, 112], thus the contra-lateral “healthy” leg should also be considered as training target. Instead of considering each leg separately, a more holistic approach regarding overall movement quality has been proposed [15, 16].

There are some limitations in this systematic review that need to be considered. (1) We included studies published only in English, (2) most of the studies had limited sample size and thus were underpowered to adjust for gender, or graft type, which may influence the reported outcomes. Thus, future investigations should assess the role of different graft types on muscle activation pattern during running, jumping and cutting/CoD tasks, in male and female ACLR participants separately, (3) there was high variation in time since surgery, ranging from ~ 4–6 months to 180 months years, and the rehabilitation protocols were not specified in most of the studies, (4) finally, the included studies investigated different running tasks such over-ground and treadmill running as well as landing tasks, such as single- and double- drop jump, vertical jump, or hop landing. Because of the heterogeneity in the methodologies and the absence of a gold standard execution protocol as mentioned above, caution is warranted regarding the interpretation of the results, (5) some studies used pooled quadriceps and hamstring muscle activity although the lateral and medial components of these muscle groups have differential actions.

## Conclusion

Patients with ACLR displayed an altered muscle activity pattern during running, jumping and cutting/CoD tasks, even though they were considered to be capable for sport return. Although there was great heterogeneity in the subject selection and study methods, the ACLR leg displayed decreased quadriceps or increased hamstrings EMG activity or both despite RTS. Simultaneous decreased quadriceps and increased hamstrings EMG activity was shown for both running and jumping/landing irrespective of graft.

The clinical relevance is that this combination, i.e. “hamstrings dominant” strategy, can serve as a protective mechanism against graft re-injury by reducing anterior shear forces at the knee. More studies are needed to establish whether there is indeed a link between the “hamstrings dominant” strategy and reduced re-injury risk.

## Supplementary Information

**Additional file 1: Table S1. **Risk of bias assessment for electromyographic running studies.** Table S2. **Risk of bias assessment for electromyographic jumping/landing studies.** Table S3. **Risk of bias assessment for electromyographic cutting/CoD studies.
