# Supplementary material for: Neuromuscular activity of the lower-extremities during running, landing and changing-of-direction movements in individuals with anterior cruciate ligament reconstruction: a review of electromyographic studies
Source: J Exp Orthop. 2023 Apr 14;10:43. doi: 10.1186/s40634-023-00603-1 (PMC10105000; doi:10.1186/s40634-023-00603-1)
Supplement: Supplementary file 1 — Additional file 1: Table S1. Risk of bias assessment for electromyographic running studies. Table S2. Risk of bias assessment for electromyographic jumping/landing studies. Table S3. Risk of bias assessment for electromyographic cutting/CoD studies. [file 40634_2023_603_MOESM1_ESM.pdf]

Table S1. Risk of bias assessment for electromyographic running studies

Study	Reporting								External validity			Internal validity													Power	Score	Quality
	1	2	3	4	5	6	7	10	11	12	13	14	15	16	17	18	20	21	22	23	24	25	26	27	Total	Rating
Einarsson et al., 2021	1	1	1	1	2	1	1	1	X	0	0	X	X	1	X	1	1	X	X	X	X	1	X	0	13	Medium
Jafarnezhadgero et al., 2021	1	1	1	1	2	1	1	1	X	0	0	X	X	1	X	1	1	X	X	X	X	1	X	1	14	Medium
Patras et al., 2012	1	1	0	1	2	0	1	0	X	0	0	X	X	0	X	1	1	X	X	X	X	X	X	0	8	Low
Patras et al., 2011	1	1	1	1	1	1	1	1	0	0	0	1	X	1	X	1	1	X	X	X	X	1	X	0	14	Medium
Patras et al., 2010	1	1	1	1	2	1	1	1	0	0	0	X	X	1	X	1	1	X	X	X	X	1	X	0	13	Medium
Patras et al., 2009	1	1	1	1	2	1	1	1	X	0	0	X	X	1	0	1	1	IC	X	X	X	IC	X	0	12	Medium

1=clear aim; 2=adequate description of outcomes; 3=description of patients' characteristics; 4=description of interventions; 5=description of potential confounders; 6=description of main findings; 7=estimates of random variability for the main outcomes; 10=probabilities for main outcomes; 11=subjects representative of population; 12=subjects asked represent population; 13=staff/facilities description; 14=subjects blinding; 15=researchers blinding; 16=data dredging; 17=adjustment for different lengths of follow-up; 18=statistical tests for main outcomes; 19=intervention compliance; 20=validity and reliability of main outcome measures; 21=cases-controls from the same population; 22=cases-controls recruited at the same time; 23=subjects randomization; 24=assignment concealed; 25=results adjusted with confounding factor; 26=adjustment for patient loss to follow-up; 27=power analysis

Table S2. Risk of bias assessment for electromyographic jumping/landing studies

Study	Reporting								External validity			Internal validity													Power	Score	Quality
	**1**	**2**	**3**	**4**	**5**	**6**	**7**	**10**	**11**	**12**	**13**	**14**	**15**	**16**	**17**	**18**	**20**	**21**	**22**	**23**	**24**	**25**	**26**	**27**	**Total**	**Rating**
Markström et al., 2022	1	1	1	1	2	1	1	1	X	0	1	X	X	1	1	1	1	1	X	X	X	1	X	0	16	Medium
He et al., 2022	1	1	1	1	2	1	1	1	X	0	X	X	X	1	X	1	1	1	X	X	X	X	X	1	14	Medium
Alanazi et al., 2021	1	1	1	1	2	1	1	1	X	0	1	X	X	1	X	1	1	1	X	X	X	1	X	1	16	Medium
Behnke et al., 2021	1	1	1	1	2	1	1	1	X	0	1	X	X	1	X	1	1	1	X	X	X	0	X	0	14	Medium
Rocchi et al., 2020	1	1	1	1	2	1	1	1	X	0	1	X	X	1	X	1	1	1	X	X	X	1	X	0	15	Medium
Alanazi et al., 2020	1	1	1	1	1	1	1	1	X	0	1	X	X	1	X	1	1	1	X	X	X	1	X	0	14	Medium
Burland et al., 2020	1	1	1	1	2	1	1	1	X	0	1	X	X	1	X	1	1	1	X	X	X	X	X	1	15	Medium
Smeets et al., 2020	1	1	1	1	2	1	1	1	X	0	1	X	X	1	X	1	1	1	X	X	X	X	X	0	14	Medium
Dashti Rostami et al., 2020	1	1	1	1	1	1	0	1	X	0	1	X	X	1	X	1	1	1	X	X	X	X	X	1	13	Medium
Dashti Rostami et al., 2019	1	1	1	1	1	1	1	1	X	0	1	X	X	1	1	1	1	X	X	X	X	1	X	1	15	Medium
Palmieri-Smith et al., 2019	1	1	1	1	2	1	1	1	0	0	X	X	X	1	X	1	1	1	X	X	X	X	X	0	13	Medium
Lessi et al., 2018	1	1	1	1	2	1	1	1	X	0	1	X	X	1	1	1	1	1	0	X	X	1	X	1	17	High
Jordan et al., 2017	1	1	1	1	2	1	1	1	X	0	0	X	X	1	X	1	1	1	X	X	X	X	X	1	14	Medium
Lessi et al., 2017	1	1	1	1	2	1	1	1	X	0	1	X	X	1	1	1	1	1	0	X	X	1	X	0	16	Medium
Melińska et al., 2015	1	1	1	1	1	1	1	1	0	0	X	X	X	1	X	1	1	1	X	X	X	0	X	0	12	Low
Nyland et al., 2014	1	1	1	1	2	1	1	1	1	0	1	X	X	1	1	1	1	X	1	X	X	1	X	0	17	High
Ortiz et al., 2014	1	1	1	1	2	1	1	1	X	0	1	X	X	1	X	1	1	1	X	X	X	0	1	0	15	Medium
Ortiz et al., 2011	1	1	1	1	2	0	0	0	X	0	1	X	X	1	X	1	1	X	X	X	X	0	X	0	10	Low
Nyland et al., 2010	1	1	1	1	2	1	1	1	1	0	1	X	X	1	1	1	1	0	1	X	X	0	X	0	17	High
Gokeler et al., 2010	1	1	1	1	1	1	0	1	X	0	1	X	X	1	X	1	1	X	X	X	X	X	X	0	12	Low
Bryant et al., 2009	1	1	1	1	2	1	1	1	X	0	1	X	X	1	1	1	1	1	X	X	X	1	X	0	16	Medium
Ortiz et al., 2008	1	1	1	1	2	0	0	1	X	0	1	X	X	1	X	1	1	X	X	X	X	0	X	0	11	Low

1=clear aim; 2=adequate description of outcomes; 3=description of patients' characteristics; 4=description of interventions; 5=description of potential confounders; 6=description of main findings; 7=estimates of random variability for the main outcomes; 10=probabilities for main outcomes; 11=subjects representative of population; 12=subjects asked represent population; 13=staff/facilities description; 14=subjects blinding; 15=researchers blinding; 16=data dredging; 17=adjustment for different lengths of follow-up; 18=statistical tests for main outcomes; 19=intervention compliance; 20=validity and reliability of main outcome measures; 21=cases-controls from the same population; 22=cases-controls recruited at the same time; 23=subjects randomization; 24=assignment concealed; 25=results adjusted with confounding factor; 26=adjustment for patient loss to follow-up; 27=power analysis

Table S3. Risk of bias assessment for electromyographic cutting/CoD studies

**Study**	**Reporting**								**External validity**			**Internal validity**													**Power**	**Score**	**Quality**
	**1**	**2**	**3**	**4**	**5**	**6**	**7**	**10**	**11**	**12**	**13**	**14**	**15**	**16**	**17**	**18**	**20**	**21**	**22**	**23**	**24**	**25**	**26**		**27**	**Total**	**Rating**
Arumugam & Hager, 2022	1	1	1	1	2	1	1	1	0	X	X	X	X	1	X	1	1	1	0	X	X	1	X		0	14	Medium
Zebis et al., 2017	1	1	1	1	2	1	0	0	X	0	1	X	X	1	X	1	1	X	X	X	X	X	X		0	11	Low
Briem et al., 2016	1	1	1	1	2	1	1	1	1	0	1	X	X	1	X	1	1	1	1	X	X	1	X		1	18	High
Coats-Thomas et al., 2013	1	1	1	X	1	1	1	1	0	0	X	X	X	1	X	1	1	1	0	X	X	0	X		0	11	Low
Ortiz et al., 2011	1	1	1	1	2	0	0	0	X	0	1	X	X	1	X	1	1	X	X	X	X	0	X		0	10	Low

1=clear aim; 2=adequate description of outcomes; 3=description of patients' characteristics; 4=description of interventions; 5=description of potential confounders; 6=description of main findings; 7=estimates of random variability for the main outcomes; 10=probabilities for main outcomes; 11=subjects representative of population; 12=subjects asked represent population; 13=staff/facilities description; 14=subjects blinding; 15=researchers blinding; 16=data dredging; 17=adjustment for different lengths of follow-up; 18=statistical tests for main outcomes; 19=intervention compliance; 20=validity and reliability of main outcome measures; 21=cases-controls from the same population; 22=cases-controls recruited at the same time; 23=subjects randomization; 24=assignment concealed; 25=results adjusted with confounding factor; 26=adjustment for patient loss to follow-up; 27=power analysis
